# Use of small needle knife in autologous fat grafting for the treatment of depressed scar: A case report

**DOI:** 10.1097/MD.0000000000009266

**Published:** 2017-12-15

**Authors:** Songjia Tang, Xiaoxin Wu, Haiyan Shen, Yuyan Wang, Jinsheng Li, Jufang Zhang

**Affiliations:** a Plastic and Aesthetic Surgery Department of Hangzhou First People's Hospital; b State Key Laboratory for Diagnosis and Treatment of Infectious Diseases, Collaborative Innovation Center for Diagnosis and Treatment of Infectious Diseases, the First Affiliated Hospital, School of Medicine, Zhejiang University, Hangzhou, P.R. China.

**Keywords:** autologous fat grafting, depressed scar, small needle knife

## Abstract

**Rationale:**

Scars always related to functional limitations, cosmetic impairment, and social and emotional problems. Clinical improvements in scar characteristics after autologous fat grafting are well described. In this paper, we present an innovative approach to treat depressed scars.

**Patient concerns:**

We presented a 29-year-old woman with multiple depressed scars in the left upper arm and near the elbow joint after trauma in childhood.

**Diagnoses:**

The patient was diagnosed as having multiple depressed scars accompanied with retraction and pain.

**Interventions:**

We used small needle knife during fat grafting to treat the depressed scar. Vancouver Scar Scale was used to assess the effect.

**Outcomes:**

Aesthetic and functional improvements were observed. Resolution of pain and improvement in scar elasticity were objectively assessable. Improvement of both clinical evaluation and patient perception was obtained.

**Lessons:**

Use of small needle knife during fat grafting is a good alternative for the treatment of depressed scars.

## 1 Introduction

Depressed scar is a common abnormality which could cause emotional, social, and behavioral problems, especially when it is located in exposed areas. Revision surgical approaches as excision and resuturing, Z-plasty, and W-plasty have been applied to treat scar in selected cases. Autologous fat grafting has proved to be efficient and safe to treat scars caused by different factors.^[1]^ Histological examination of treated area showed tissue reconstruction similar to the original.^[2]^

Various techniques have been developed to achieve a better treatment effect of fat grafting. However, a standard procedure has not been put forward. In our experience, a compatible environment is important for adipose tissue to survive and proliferate. Classic procedure of fat grafting with blunt cannulas failed to perform quickly under scar tissue and could not provide a released subcutaneous environment. We used small needle knife to release scar bundles in superficial and deep planes before fat grafting. Vancouver Scar Scale (VSS) questionnaire was used for clinical evaluation of surgical outcomes and to prove the efficacy of fat grafting.

## 2 Case presentation

A 29-year-old female, with depressed scar in the left upper arm, is reported. The patient had retractile and painful scars near the elbow joint, although joint mobility was not limited. The scars were caused by trauma 20 years ago. The largest was approximately 1.6 cm × 2.0 cm in size and 0.8 cm in depth; the smallest was about 0.8 cm × 3.0 cm in size and 0.5 cm in depth. The patient had not received any treatment before.

The patient underwent clinical assessment and routine preoperative examinations. Written informed consent was obtained from the patient.

**Figure 1:**
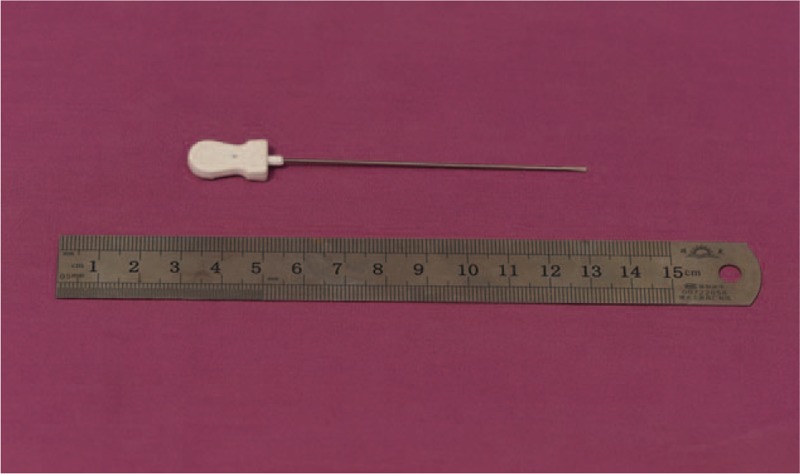
Small needle knife: 1.0 mm in diameter, 80 mm in length.

The surgical procedure was performed under local anesthesia. Abdomen was prepared as donor site and infiltrated with anesthetic solution (500 mL saline solution, 20 mL of lidocaine 0.02 mg/mL, and 0.5 mL epinephrine 1 mg/mL). Adipose tissue was harvested using the Coleman technique. The collected lipoaspirate was then sedimented for grafting. Under local anesthesia, small needle knife (Fig. 1) was inserted under skin to release scar adhesion. Crossed radiating passages were made to sufficiently break the subcutaneous fibrosis and form an ideal form of web for grafting. Fat was transferred in a 1-mL syringe Luer lock and injected into the released and loosened fibrous structure. Constant amount of fat was laid at the dermal-hypodermal layer. Digital pressure with soft gauze was applied immediately after placement to make local appearance flat and smooth, and minimize contour irregularities. Gauze dressing was covered in the treated area for 1 week and the patient was told to avoid pressure and friction to limit displacement of fat infiltration. Incisions in the donor site were sutured with nylon 5/0. Elastic-compressive dressing was applied in the donor site to prevent hematoma.

**Figure 2:**
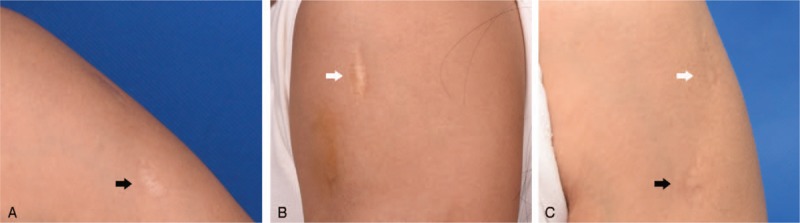
A 29-year-old female had 2 depressed scars in the left upper arm. The scars were caused by trauma 20 years ago. One was approximately 0.8 cm × 3.0 cm in size, 0.5 cm in depth (white arrow); the other was about 1.2 cm × 2.5 cm in size, 0.3 cm in depth (black arrow). Color of scars was improved and depressions were corrected. (A, B) Preoperative; (C) 3 months after surgery.

**Figure 3:**
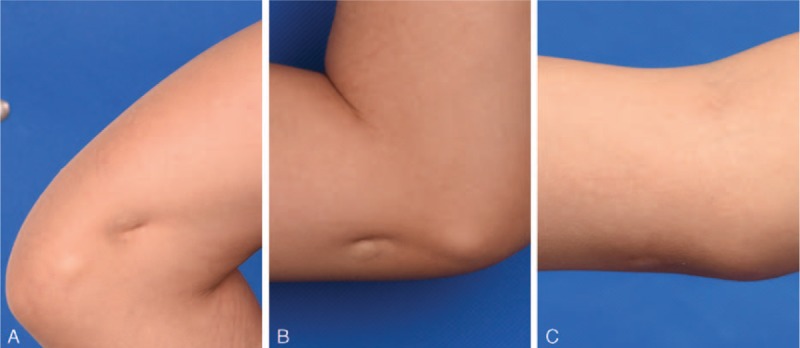
The same patient had 1 retractile and painful scar near the elbow joint, although joint mobility was not limited. The scar was about 1.6 cm × 2.0 cm in size, 0.8 cm in depth. Retraction and pain were improved after surgery. (A, B) Preoperative; (C) 3 months after surgery.

Clinical assessment of scars with the VSS was performed before surgery and at 3 months of follow-up. Scar retraction, pain, and passive and active joint function were evaluated. The VSS evaluation of the physician was not known to the patient. Quality improvement was shown both aesthetically and functionally. Volume deficits were refilled with fat grafting, and excellent cosmetic results were achieved (Fig. 2, Fig. 3). Skin became softer and more extensible. Pain and scar elasticity were improved after 3 months follow-up (Table 1).

**Table 1:**
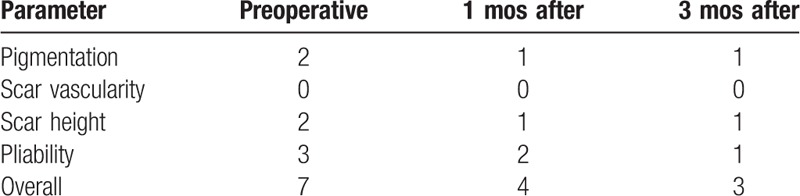
Vancouver Scar Scale evaluation.

## 3 Discussion

Scar tissue can cause cosmetic impairments, functional limitations, pain, itch, and psychological problems.^[3]^ Common therapies include surgical, medical, and conservative procedures, such as excision and resuturing, elastic-compressive garments, topical silicone sheets, corticosteroid injection, laser therapy, and radiotherapy, which may achieve positive but partial effects.^[1]^ Autologous fat grafting, first described by Neuber in 1893 and refined by Coleman^[4]^ in 1992, has been introduced as a promising treatment option for scar tissue-related symptoms with limited side effects. It is considered fat graft could improve scar quality by volume-increasing effect,^[5,6]^ and mitigate pain by changing the microenvironment and secretion of substances by mesenchymal cells.^[7]^ Histological examinations showed dermal and dermohypodermic quality were improved with new collagen deposition and local neoangiogenesis in the scar area.^[2,5]^ There have been different harvesting procedures, processing techniques, and reinjection methods to optimize fat graft survival.^[8]^ However, a universal protocol has not been chosen, and resorption of volume after autologous fat grafting remains a challenge.^[3]^

Small needle knife is 1 mm in diameter and 10 cm in length, with one knife end. It is widely used to mitigate arthralgia and arthritis through adhesiolysis of tissue in traditional Chinese medicine.^[9]^ During fat grafting, small needle knife was inserted to release scar fibrosis and adhesion, which significantly decreased the resistance to the sliding of fat injection devices under the scar. Crossed web-form passages were made for even distribution and better survival of adipose tissue, which help increase rooting, and minimize the possibility of forming cysts.^[2]^ Moreover, we also considered that a sharp small needle knife, similar to a “needling” procedure used in aesthetic medicine, could stimulate new collagen deposition and remodeling of fibrous tissues. Compared with angiographic needle, small needle knife is harder in texture, but less sharp at the end, which decreases edema and bleeding of treated area. Injecting with blunt cannulas after scar adhesiolysis could effectively prevent intravascular fat injection.

## 4 Conclusions

In the reported case, the patient's retractile skin was loosened, and pain perception was decreased. Volume deficits were improved and the effect continued at 3 months of follow-up. Differences of appearance between 2 upper arms were decreased. At this writing, a future follow-up assessment is planned. Hence, we think that our surgical method using small needle knife in autologous fat grafting is a good alternative for the treatment of depressed scars, considering multiple advantages and excellent results obtained.
